# Effect of weight loss program using prebiotics and probiotics on body composition, physique, and metabolic products: longitudinal intervention study

**DOI:** 10.1038/s41598-024-61130-2

**Published:** 2024-05-14

**Authors:** Nayera E. Hassan, Sahar A. El-Masry, Salwa M. El Shebini, Nihad H. Ahmed, Nayra Sh. Mehanna, Mai Magdy Abdel Wahed, Darine Amine, Adel Hashish, Mohamed Selim, Mahmoud A. S. Afify, Khadija Alian

**Affiliations:** 1 Biological Anthropology Department, Medical Research and Clinical Studies Institute, National Research Centre, 33 El-Buhouth St., Dokki, Giza, 12622 Egypt; 2 Nutrition and Food Science Department, Nutrition and Food Science Institute, National Research Centre, Giza, Egypt; 3 Dairy Science Department, Nutrition and Food Science Institute, National Research Centre, Giza, Egypt; 4 Clinical and Chemical Pathology Department, Medical Research and Clinical Studies Institute, National Research Centre, Giza, Egypt; 5 Children with Special Needs Department, Medical Research and Clinical Studies Institute, National Research Centre, Giza, Egypt; 6 Researches and Applications of Complementary Medicine Department, Medical Research and Clinical Studies Institute, National Research Centre, Giza, Egypt

**Keywords:** Body composition, Microbiota, Prebiotics, Probiotics, Microbiology, Diseases, Health care

## Abstract

The relationship between gut microbiota and obesity has recently been an important subject for research as the gut microbiota is thought to affect body homeostasis including body weight and composition, intervening with pro and prebiotics is an intelligent possible way for obesity management. To evaluate the effect of hypo caloric adequate fiber regimen with probiotic supplementation and physical exercise, whether it will have a good impact on health, body composition, and physique among obese Egyptian women or has no significant effect. The enrolled 58 women, in this longitudinal follow-up intervention study; followed a weight loss eating regimen (prebiotic), including a low-carbohydrate adequate-fiber adequate-protein dietary pattern with decreased energy intake. They additionally received daily probiotic supplements in the form of yogurt and were instructed to exercise regularly for 3 months. Anthropometric measurements, body composition, laboratory investigations, and microbiota analysis were obtained before and after the 3 months weight loss program. Statistically highly significant differences in the anthropometry, body composition parameters: and obesity-related biomarkers (Leptin, ALT, and AST) between the pre and post-follow-up measurements at the end of the study as they were all decreased. The prebiotic and probiotic supplementation induced statistically highly significant alterations in the composition of the gut microbiota with increased relative abundance of Lactobacillus, Bifidobacteria, and Bacteroidetes and decreased relative abundance of Firmicutes and Firmicutes/Bacteroidetes Ratio. Hypo caloric adequate fiber regimen diet with probiotics positively impacts body composition and is effective for weight loss normalizing serum Leptin and AST.

## Introduction

Obesity presents a worldwide problem that prevails in almost all countries, and affects all socioeconomic classes, all ages, all races and both genders^1^***.*** White adipose tissue of obese persons is in a persistent low-grade inflammation, causing systemic complications^2,3^***.***

The adipose tissue macrophage is actually the most important cell responsible for obesity-related inflammation. Macrophages surround dead adipocytes forming the *crown-like structures* (CLS). These cells in association with enlarged adipocytes initiate the peculiar obesity-related subclinical pro-inflammatory cascade^2,4,5^.

Gut microbiota is defined as the community of microorganisms that live in the gastrointestinal tract. Microorganisms in the human gut are complex and dynamic with a marked impact on the host`s normal homeostasis as well as disease conditions. Various factors contribute to building up the gut microbiota population starting from birth and throughout life. Diet is considered one major factor^6^***.*** Dietary probiotics alter gut microbiota and can be effective for both inducing weight loss and preventing weight regain^7^***.***

The gut Microbiome presents an eminent role in health maintenance, promoting the metabolism of indigestible dietary elements and the essential synthesis of some vitamins, contributing to the maturation and the evolving memory of the immune system, and hindering pathogen colonization^8^*.* Changes in the composition of gut microbiota affect gut health and also may affect the health of other body tissues by immune mechanisms^9,10^.

The human gut microbiota consists mainly of two dominant phyla, *Bacteroidetes and Firmicutes*. These two phyla represent 90% of the total community or even more. Other less dominant phyla include *Proteobacteria*, *Actinobacteria*, and *Verrucomicrobia*^8,11^.

This composition tends to stay constant despite possible perturbations, and it can rapidly return to its basic formation. Firmicutes/Bacteroidetes (F/B) proportion was higher in obese people than lean people, and tended to decrease with weight reduction^12^.

It has been noticed that Firmicutes were more effective than Bacteroidetes in extracting energy from food, thus promoting more efficient calorie absorption with subsequent weight gain. Thus, recently, the Firmicutes/Bacteroidetes ratio has been frequently considered a probable obesity hallmark^13^.

The relationship between obesity and microbiota is complex, both affecting susceptibility to obesity, as well as affecting the amounts and properties of microbiota because of the changing environment with obesity^6^.

Imbalance in the gut microbiota may contribute to overweight and obesity as scientific evidence suggests, and despite the complexity of adiposity pathogenesis, manipulations to enable a balanced microbiotic status, could give a potential therapy for obesity^14^.

The aim of this study was evaluation of the effect of hypo caloric adequate fiber regimen, physical exercise, and probiotic supplementation on the improvement of health, body composition, and physique among obese Egyptian women.

## Subjects and methods

### Subjects

This was a longitudinal follow-up intervention study that included 58 obese Egyptian women with a mean age of 41.62 ± 10.70 years. It was carried out at the Medical Research Excellence Centre (MERC), National Research Centre. It was part of a cross-sectional survey of a project funded by the National Research Centre (NRC) Egypt, 2019–2022 entitled ‘‘Gut Microbiota in Obesity and Metabolic Syndrome among obese women: Interactions of the Microbiome, Epigenetics, Nutrition, and Probiotic Intervention” (12th Research Plan of the NRC).

The cross-sectional survey included 82 obese women with age ranged from 25 up to 60 years. Fifty-eight women only of them completed the longitudinal follow-up intervention study.

The study protocol conformed to the ethical guidelines of the 1975 Declaration of Helsinki and was approved by the Ethics Committee of the NRC (Approval no 19/236). All participants provided their informed consent.

The participants of the study were enrolled in a weight loss program. Those enrolled were initially obese and had a mean BMI of 38.32 ± 4.01 kg/m^2^.

Subjects with conditions that may impact gut microbiota (gastrointestinal, autoimmune, and metabolic diseases and medications, particularly antibiotics) were not included in the study.

The enrolled women followed a weight loss program which included a hypo caloric adequate fiber (prebiotic) eating regimen (a low-carbohydrate adequate-protein and fiber dietary pattern with reduced energy intake); they additionally received daily probiotic supplement in the form of yogurt and were instructed to exercise regularly for 3 months.

## Methods

For each participant, anthropometric measurements, body composition, laboratory investigations, and microbiota analysis were obtained before and after the 3 months weight loss program.

### Anthropometric measurements

Body weight, height, waist and hip circumferences, and skin fold thickness at 4 sites (triceps, biceps, subscapular, and abdominal) were measured, following the recommendations of the “International Biological Program”^15^.

Body weight (Wt) was determined to the nearest 0.01 kg using a Seca Scale Balance, with the woman wearing minimal clothes and with no shoes.

Body height (Ht) was measured to the nearest 0.1 cm using a Holtain portable anthropometer.

Waist circumference (WC) was measured at the midpoint between the lower curvature of the last fixed rib and the superior curvature of the iliac crest, with the woman in an upright standing position and their arms alongside the body, feet together, and abdomen relaxed.

Hip circumference was measured at the maximum extension of the buttocks measuring the largest diameter above the symphysis pubis overlapping the apex of the buttocks.

Skin fold thicknesses were measured at the left side using Holtain skin fold caliper and approximated to the nearest 0.1 mm.

The previously mentioned measurements were used to calculate the following parameters: Body Mass Index [BMI: weight (in kilograms) divided by height (in meters squared)], waist/hip ratio WHR (cm/cm), waist/height ratio WHTR (cm/cm), the peripheral adiposity index [sum of triceps and biceps skin fold thicknesses (mm)] and central adiposity index [sum of subscapular and abdominal skin fold thicknesses (mm)]. The participating women were all chosen as obese; as their BMI was ≥ 30 kg/m^2^.

#### Body composition

Body composition was assessed using the TANITA Body Composition Analyzer. As specified by the manufacturer (Tanita Body Composition Analyzer-MC-780 MA III), the unit was calibrated before testing. The participating woman stood on the footboard of the device, while she was holding the 2 handles carefully, each by one hand at the same time. By using her sex, age, weight, and height, approximated to the nearest unit, the percentage body fat (Fat (%): an estimate of the fraction of the total body mass that is adipose tissue), Fat Mass in Kg (FM: an estimate of the fraction of the total body weight that is adipose tissue), Fat-Free Mass in Kg (FFM: an estimate of the fraction of the total body weight that is not adipose tissue ), body water content ( in liter), and Basal Metabolic Rate (BMR in kilo calories: the rate at which the body uses energy, while at rest, to maintain vital functions such as breathing and keeping warm), were measured.

#### Blood sampling and laboratory investigations

In the morning, venous blood samples were drawn from the participating women, using venipuncture. Biochemical parameters were performed on sera that were stored at − 70 °C until used for assessment of Short Chain Fatty Acids (SCFA), Leptin, and liver enzymes: Aspartate aminotransferase (AST) and Alanine aminotransferase (ALT). All were done in the laboratory of “The Medical Excellence Research Center MERC” which is a part of “NRC”, Egypt.

Human Short Chain Fatty Acids (SCFA) were assessed in serum using Enzyme-Linked Immunosorbent Assay (ELISA) kits; Catalog Number: MBS7269061 according to the method described by den Besten et al.^16^.

The assay of human Leptin in serum was performed by the ELISA method, using kits of BioLegend Inc. (San Diego, California, USA), according to the method of Considine et al.^17^.

Serum concentrations of AST and ALT were determined using the automated clinical chemistry analyzer Olympus AU 400 analyzer. https://www.mybiosource.com

#### Microbiota analysis

To characterize the effects of the weight loss program eating plan, physical exercise, and probiotic supplement on the gut microbiota of the study participants, fecal samples were obtained before and after the intervention, gene sequence analysis was performed, and individual variations of gut microbiota were compared.

The proportion of Lactobacillus and Bifidobacteria; and Firmicutes/Bacteroidetes ratio strains were assessed in the stool of all participants by using the real-time PCR (Polymerase Chain Reaction). Specimen collection and preparation: The stool was collected by defecation in a plain sterilized container and allowed to be frozen. *Specimen Storage and Preparation:* The stool was frozen at − 20°. The primers and probes were used to detect Bifidobacteria spp. and Lactobacillusspp.; Firmicutes spp. and Bacteroidetes spp., were based on 16S rRNA gene sequences retrieved from the National Center for Biotechnology Information databases using the Entrez program^18^. Reagents provided by kits: DNA Extraction Kit. Assay procedure: DNA extraction: The QIAamp DNA Stool Minikit (Qiagen) was used to extract DNA from one gram of fresh or frozen stool sample according to the manufacturer’s instructions. Bacterial quantification by real-time PCR was done.

### Intervention phase

All the participants were provided with the weight loss program eating plan, physical exercise, and probiotic supplement for 3 months:

1. A dietary modification plan was applied; under the supervision of a dietary consultant; using different regimens aiming to correct the wrong food habits and to supply patients with deficient nutrients (all were hypo caloric adequate fiber regimens). A typical daily meal plan is presented in Table 1**.** The prescribed regimens aimed to decrease the caloric intake of the patients and increase the fiber and protein content of the diets. To reach this goal the regimens were rich in whole grain products, beans, legumes, and nuts, dark-colored vegetables, and fruits like bananas and guava. Also it contain low-fat dairy products, cottage cheese, egg, skinless poultry and low-fat meat and fish. The mean calories supplied by the regimen was 48.83% of the RDA, while it gave 57.67 and 41.48% of the RDA of the fat and carbohydrate. However, it supplied 88.38 and 95.04% of the RDA of the protein and fiber.”
2. Performing adequate aerobic exercise, pre-participation evaluation, designing the program, patient education, specific programs for each age group; and daily classes for health and fitness (by video).
3. In addition, they were provided with probiotic supplementation: 100 g from a dietary supplement product (fermented milk in the form of Yogurt) which contained probiotic strains (10^8^/g). It was taken orally once a day for 3 months. Probiotic strains were obtained from a capsule of probiotic “GNC ultra probiotic complex 100” which contains a mix of probiotic strains, isolated by the National Research Centre (NRC) probiotics lab. The probiotic component of the product used in the study contained a blend (one fermented milk cup contained 100 g, 10 × 10^9^ CFU) of proprietary strains of Lactobacillus acidophilus CUL60, Lactobacillus acidophilus CUL21, Lactobacillus acidophilus NCFM, Bifidobacterialactis HNO19, Bifidobacteriaanimalissupsplactis CUL34, and Bifidobacteriabifidum CUL20 was purchased from GNC Ultra Probiotic Complex (UK)

**Table 1:** Mean ± SE of nutrients intake of the studied women during dietary therapy.

Nutrient intake	Dietary Regimen followed for 3 months
	Mean ± SE % of RDA
Energy (Cal)	1074.29 ± 15.34
RDA: 2200	48.83
Protein (g)	44.19 ± 5.90
RDA:50	88.38
Fat (g)	44.41 ± 2.31
RDA:70	57.67
Carbohydrate (g)	124.46 ± 6.29
RDA:300	41.48
Dietary fiber (g)	23.76 ± 0.27
RDA:25	95.04

#### Follow-up Screening phase

Collection of the follow-up data for all participants to evaluate the effect of hypo caloric adequate fiber regimen, physical exercise, and probiotics on the improvement of health every month was performed in the form of medical advice under the supervision of a professor of internal medicine. At the end of the program, the following investigation was done which included anthropometric, body composition, laboratory analysis, and gut microbiota composition changes.

### Statistical analysis

Data were analyzed using the Statistical Package for Social Sciences (SPSS/Windows Version 18, SPSS Inc., Chicago, IL, USA). The normality of data was tested using the Kolmogorov–Smirnov test. The data were normally distributed. So, the parametric tests were used. The results of the parametric data were expressed as mean ± SD, and mean differences were considered significant at *p* < 0.05.

The various parametric variables of the pre and post-intervention were analyzed and compared using Paired t-test. To explore associations between the gut microbiota, body composition, and metabolic products, Pearson’s correlation analyses were performed. Percentage of changes in the different variables between pre and post-intervention phase were calculated ad drawn using the EXCL program.

### Ethics approval and consent to participate

A written informed consent was obtained from all participants after being informed about the purpose of the study. This research paper was derived from a cross-sectional survey of a project funded by National Research Centre (NRC) Egypt, 2019–2022 entitled ‘‘Gut Microbiota in Obesity and Metabolic syndrome among obese women: Interactions of the Microbiome, Epigenetics, Nutrition and Probiotic Intervention” (12th Research Plan of the NRC). The study protocol was conformed to the ethical guidelines of the 1975 Declaration of Helsinki and was approved by the Ethics Committee of the National Research Centre (NRC) Egypt (Approval no 19/236).

## Results

In this longitudinal follow-up interventional study, the effects of the probiotic supplementation on the diversity and richness of gut microbiota and associations of bacterial species together with measuring body composition and obesity biomarkers have been evaluated among 58 obese Egyptian women participating in a weight loss program.

The mean BMI of the study participants was 38.32 + 4.01 kg/m^2^ and the average age was 47.4 years. The participating women were recruited in the weight loss program at the beginning of the study and followed a hypocaloric, adequate-protein, and adequate fiber regimen. The probiotic supplementation used in the study contained Lactobacillus acidophilus and Bifidobacteria strains. Blood and fecal samples were obtained and body composition, anthropometry, and metabolic parameters were measured at the start and at the end of the study time, the three-month interval.

The results indicated statistically highly significant differences in most of the body composition parameters: body weight, BMI, waist and hip circumstances, Waist/Height ratio (WHTR), subscapular and abdominal skin fold thickness, central adiposity index, fat percentage, fat mass, free fat mass, water content, BMR (Table 2) and obesity-related biomarkers (Leptin, ALT, and AST) (Table 3) between the pre and post follow up measurements at the end of the study (three-month prebiotic and probiotic intervention); as they were all decreased at the end of the study. While, TSF, BSF, and SCFA demonstrated a statistically insignificant decreasing trend at the end of the study.

**Table 2:** Comparisons between pre and post follow up anthropometric measurements and fat distribution among 58 obese females.

Variable	Pre		Post		*P*
	Mean	+ SD	Mean	+ SD	
Weight (Kg)	91.11	15.03	89.54	13.75	0.000**
BMI (Kg/m^2^)	38.32	4.01	37.68	3.40	0.000**
WC (cm)	111.70	11.31	110.64	13.48	0.000**
Hip C (cm)	123.60	7.80	115.20	11.21	0.001**
WHR (cm/cm)	0.89	0.08	0.95	0.05	0.233
WHTR(cm/cm)	0.73	0.06	0.72	0.08	0.000**
Skin fold thickness (mm)					
Triceps	29.08	6.23	22.50	5.69	0.071
Biceps	28.75	5.26	26.00	4.38	0.330
Subcapular	32.33	8.59	21.50	2.74	0.022*
Abdominal	41.50	3.42	30.50	3.84	0.000**
Peripheral obesity Index	57.83	10.42	48.50	9.81	0.080
Central obesity Index	73.83	10.88	52.00	2.97	0.000**
Body composition					
Fat%	46.13	2.56	45.51	3.11	0.000**
Fat mass	42.38	8.95	41.07	8.73	0.000**
FFM	48.87	6.22	48.52	5.84	0.000**
Water	35.77	4.55	35.54	4.26	0.000**
BMR	6386.91	846.36	6322.09	784.00	0.000**

*Significant difference at *p* < 0.05, **Highly significant difference at *p* < 0.01.

**Table 3:** Comparisons between pre and post follow up laboratory investigations among 58 obese females.

Variable	Pre		Post		*P*
	Mean	+ SD	Mean	+ SD	
SCFA	17.41	16.78	6.76	1.88	0.134
Leptin	18,687.93	7145.83	1583.45	1174.11	0.027*
ALT	25.84	4.00	19.26	3.80	0.000**
AST	25.90	8.58	11.95	5.16	0.006**
Microbiota					
Log Lactobasillus	5.74	0.59	6.22	0.90	0.000**
Log Bifidobacterium	6.47	0.70	6.73	0.66	0.000**
Log Bacteroidetes	13.31	1.18	13.40	1.23	0.000**
Log Firmicutes	8.49	1.17	8.31	1.04	0.000**
Firmicutes/Bacteroid Ratio	0.65	0.13	0.63	0.13	0.000**

*Significant difference at *p* < 0.05, **Highly significant difference at *p* < 0.01.

The prebiotic and probiotic supplementation created statistically highly significant alterations in the gut microbiota composition at the end of the study (Table 3). Increments in Lactobacillus, Bifidobacteria Bacteroidetes relative abundance were observed following prebiotic and probiotic intervention, while the Firmicutes relative abundance and Firmicutes/Bacteroidetes Ratio at the end of the study were significantly lower.

**Figure 1:**
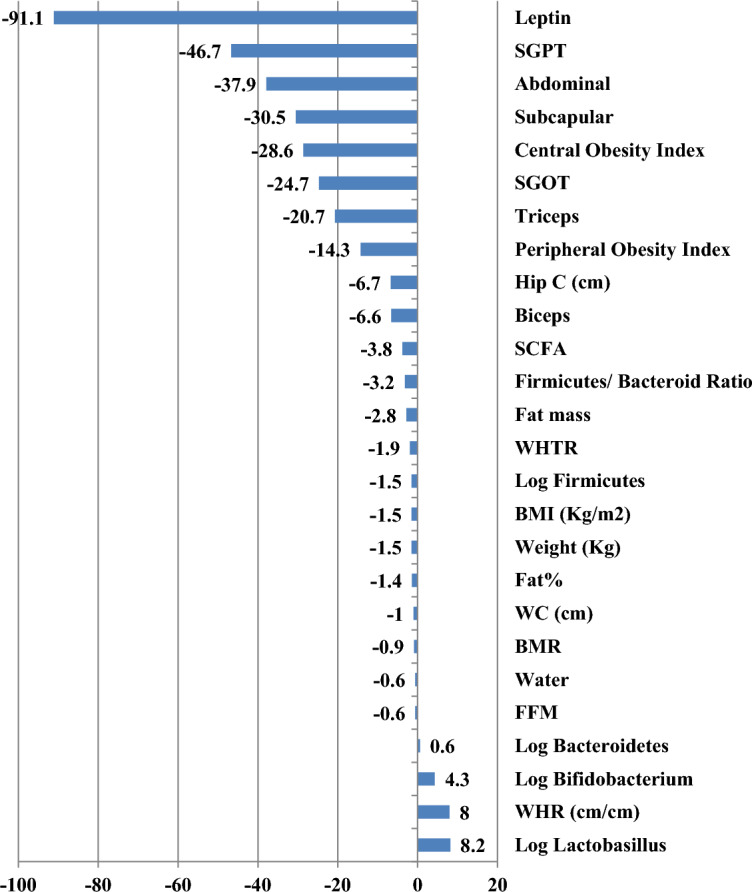
Changes %of the different variables between pre and post follow up.

The percentages of changes in the different variables after the intervention (Fig. 1), revealed that the most decreased variables were Leptin (↓91.1%), followed by AST (↓46.7%), abdominal (↓37.9%) and subscapular skin fold thickness (↓30.5%), central obesity index (↓28.6%), ALT (↓24.7%), TSF (↓20.7), and peripheral obesity index (↓14.3%). On the other hand, there were increased values of some microbiota: Lactobacillus (↑8.2%), Bifidobacteria (↑4.3%) and Bacteroidetes (↑0.6%), while Firmicutes (↓1.5%), and Firmicutes/Bacteroidetes Ratio (↓3.2%) were decreased.

Table 4 shows the Pearson’s correlation coefficient between Firmicutes/Bacteroidetes Ratio and the anthropometric, fat distribution, body composition, and laboratory investigations before and after the intervention. After supplementation in the obese women, the results revealed persistence of the negative significant correlation between the ratio and the body WT, WC, WHR, WHTR, subscapular SF, central adiposity index, FFM, water content, BMR, and SCFA. The negative correlations between the ratio and each of BMI, hip C, FM, Leptin, and AST after the intervention became significant. While, the significant negative correlations between the ratio and each of the TSF, BSF, and peripheral obesity index disappeared after the intervention.

**Table 4:** Spearman’s correlation between Firmicutes/Bacteroid Ratio among pre and post follow up anthropometric measurements and fat distribution of obese females.

Variable	Firmicutes/Bacteroid Ratio			
	Pre		Post	
	r	*p*	r	*p*
Weight (Kg)	− 0.304	0.020*	− 0.368	0.004**
BMI (Kg/m^2^)	− 0.156	0.241	− 0.261	0.048*
WC (cm)	− 0.587	0.000**	− 0.565	0.000**
Hip C (cm)	0.124	0.353	− 0.521	0.001**
WHR (cm/cm)	− 0.736	0.000**	− 0.454	0.006**
WHTR(cm/cm)	− 0.479	0.000**	− 0.473	0.001**
Skin fold Thickness (mm):				
Triceps	− 0.602	0.000**	− 0.275	0.157
Biceps	− 0.452	0.000**	0.165	0.401
Subcapular	− 0.774	0.000**	− 0.800	0.000**
Abdominal	− 0.113	0.398	0.061	0.759
Peripheral Obesity Index	− 0.573	0.000**	− 0.086	0.664
Central Obesity Index	− 0.536	0.000**	− 0.660	0.000**
body composition:				
Fat%	0.021	0.878	− 0.13	0.332
Fat mass	− 0.245	0.064	− 0.312	0.017*
FFM	− 0.386**	0.003**	− 0.408	0.001**
Water	− 0.385**	0.003**	− 0.406	0.002**
BMR	− 0.366**	0.005**	− 0.402	0.002**
Lab.				
SCFA	− 0.272*	0.039*	0.628	0.000**
Leptin	− 0.220	0.097	0.275	0.037*
ALT	0.299*	0.023*	0.150	0.261
AST	− 0.246	0.063	0.288	0.029*

*Significant difference at *p* < 0.05, **Highly significant difference at *p* < 0.01.

## Discussion

In clinical trials, dietary intervention has often led to a significant change in the diversity and abundance of different microbiota species in the human gut, which directed the attention to a possible novel mode of management of different disorders related to or correlated with gut bacteria, notably obesity. Human gut microbes are major contributors to body metabolism and have caught the attention as potential sources of new therapeutic strategies.

In the last few decades, the gut microbiota has been considered an additional factor favoring fat storage, insulin resistance, and weight gain^19^, as microbiota is involved in the control of energy balance, hunger, and satiety via gut peptide signaling, through hormonal effects in the blood or by directly regulating the nervous system. The appropriate balance of these modulatory peptides may be disrupted if the microbiota composition is changed^20^.

In the current study, the effect of an adequate fiber diet (prebiotics) together with a probiotic supplement showed a significant increase in beneficial bacteria with a decreased F/B ratio, together with changes in almost all measured parameters. Weight, body composition, and laboratory parameters were almost all significantly decreased after the intervention, taking into consideration the decrease in fat-free mass including muscle mass together with the decrease in water content that seems to be an at least early side effect of weight reduction ^21^. The lean body (fat-free mass) contains the larger amount of water among body compartments; hence it is expected to decrease with reducing muscle mass. This observation indicates the importance of adequate protein intake and possibly a resistance exercising program for maintaining muscle bulk during weight loss.

Comparatively, Sergeev et al.^22^, a placebo-controlled interventional study, evaluated the effects of synbiotic supplements on the diversity richness of gut microbiota and the associations of body composition and obesity markers with microbial species showing that low-carbohydrate, high-protein, diet is effective for weight reduction and normalizing obesity-associated metabolic defects. On the other hand, they showed no difference in body composition parameters, taking into consideration the smaller sample size (20 subjects).

On the contrary, previous studies of Raoult^23,24^, have claimed that increased ingestion of probiotics may promote weight gain by altering the intestinal microbes.

In the current study, a promising overall reduction of obesity indices, associated with a reduction of the F/B ratio and Firmicutes levels was noticed; that might refer to the claimed role of these microbes in energy extraction and contribution to obesity^25^.

Firmicutes are known to increase energy extraction by increasing the available fat for intestinal absorption, also the possible role of Butyrate they produce by fermenting carbohydrates, giving an additional source of energy that may add to the overall fuel obtained from food and made available to the circulation ^26,27^.

In a review article by FabienMagne et al.^28^, the evidence suggesting a connection between obesity and changes in the Firmicutes/Bacteroidetes ratio was not satisfactory.

In some studies, obesity has been linked to a specific profile of the intestinal microbiota, including an increase in the F/B ratio^25,29^. Also, significant associations were detected between the increase of some microbial types and obesity; e.g. Lactobacillus^30^, while other types were associated with lean status, mainly those belonging to the *Bifidobacteria* genus^31,32^.

On a scientific basis, these results are generally supported by the assumed effective mechanisms of pro-/synbiotic supplementation preventing weight gain. It may suppress inflammation^33^***,*** strengthen the intestinal mucosal barrier^34^**,** and regulate the activity of intestinal enzymes^35^**,** as well as immune and neuroendocrine functions^36^; thereby affecting energy storage^37^***,*** and expenditure^38^*.*

In the present study, a significant increase in beneficial bacteria, lactobacillus, and Bacteroidetes abundance was noticed after the intervention with pro and prebiotic supplements (the adequate fiber diet), referring with the obesity improvement to the possible role of such supplementation and eating regimen as obesity management.

Bifidobacteria is one of the major probiotics tested in human clinical trials^39^*.* When Bifidobacterialactis HN019 was introduced to obese patients with metabolic syndrome, an overall positive effect including weight reduction, decrease in blood lipids, and in some inflammatory markers was noticed. Daily consumption of probiotics showed a significant reduction in BMI, LDL, and total cholesterol compared to the control group^40^.

In some trials, Bifidobacteria was used in combination with other probiotic strains, including Lactobacillus. When a mixture of Bifidobacteriainfantis, Streptococcus thermophiles, Enterococcus faecalis, Bifidobacteriabreve, and Lactobacillus acidophilus was administered for eight weeks in the form of probiotic yogurt, body weight, and BMI were significantly diminished^41^*.*

Leptin, a hunger-inhibiting hormone secreted mainly by adipocytes and enterocytes^42^, together with AST enzyme (that may increase because of obesity-associated steatosis^43^ were the most affected parameters after this current intervention, Leptin being reduced in most of the patients, while AST being reduced in nearly half of the patients. Clinical studies have elucidated the possible role of symbiotic supplementation on obesity biomarkers including Leptin. In a randomized double-blind control study by SaeidehVafa et al.^44^, synbiotic supplementation decreased serum Leptin in overweight women diagnosed with breast cancer.

The current intervention has decreased the energy availability both in the intestine by giving lower calories, together with altering the microbiota with less energy production and extraction, and consequently in the circulation with less available calories to both liver and adipose tissue, and with body obligation to use fat stores to compensate for the induced caloric deficiency.

In contrast to many other studies^13,45–48^, unexpectedly, the current study shows a negative correlation between the F/B ratio and obesity, including anthropometry, body composition, and laboratory markers, that was noticed before and after the intervention, when it just changed to be significant in some parameters (e.g.: BMI &FM), insignificant in others (e.g.: TSF, BSF), and persisted to be significant in most of the parameters (e.g.: WT, WC, WHR, FFM, and BMR), meaning it’s been decreased in for example higher weights and vice versa, though, it is insignificant for most of the indices as explained.

Dietary proteins have been used for years to treat obesity. Body weight loss is beneficial when it concerns fat mass, but loss of fat-free mass—especially muscle might be detrimental. This occurs because protein breakdown predominates over synthesis, thus administering anabolic dietary compounds like proteins might counter fat-free mass loss while allowing for fat mass loss^49^. Accordingly, data of the current study demonstrated minimum loss in the FFM (− 0.6 kg) compared to the loss in the fat mass that reached − 2.8 k.”

### The limitation point

The restricted sample size was due to limited financial support from our institute. So, our future plan is to do another study on a wider scale involving different sectors of our community. Further, we will also study the effect of physical activity and probiotics supplementation separately, and compare them regarding their effect on weight loss.

## Conclusion

The findings obtained demonstrated that the hypo caloric adequate protein adequate fiber regimen diet with probiotic supplementation and regular exercise are effective for weight reduction and normalizing obesity-associated higher levels of Leptin, ALT, and AST, with a significant impact on body weight and body composition of subjects participating in this weight reduction program**.**

## Data Availability

The datasets used and/or analyzed during the current study are available from the corresponding author on reasonable request, after taking the permission of our institute “National Research Centre”.

## Abbreviations

ALT: Alanine amino-transferase
AST: Aspartate amino-transferase
BMI: Body Mass Index
BMR: Basal metabolic rate
BSF: Biceps skin fold thickness
ELISA: Enzyme linked immunosorbent assay
FFM: Fat free mass
FM: Fat mass
HC: Hip circumference
Ht: Body height
MERC: Medical Research Excellence Centre
NRC: National Research Centre
SCFA: Human short chain fatty acids
SF: Skin fold thickness
SPSS: Statistical package for social sciences
TSF: Triceps skin fold thickness
WC: Waist circumference
WHR: Waist/hip ratio
WHTR: Waist/height ratio
Wt: Body weight
